# Comparison of Schlemm's Canal Morphology Parameters Between Propensity Score–Matched Primary Open-Angle Glaucoma and Exfoliation Glaucoma

**DOI:** 10.1167/iovs.65.2.15

**Published:** 2024-02-07

**Authors:** Aika Tsutsui, Teruhiko Hamanaka, Sachiko Kaidzu, Kanae Kobayashi, Nobuo Ishida, Toshio Kumasaka, Masaki Tanito

**Affiliations:** 1 Department of Ophthalmology, Shimane University Faculty of Medicine, Izumo, Japan; 2 Department of Ophthalmology, Japanese Red Cross Hospital Medical Center, Tokyo, Japan; 3 Department of Ophthalmology, Ishida Eye Clinic, Niigata, Japan; 4 Department of Pathology, Japanese Red Cross Hospital Medical Center, Tokyo, Japan

**Keywords:** schlemm's canal, schlemm's canal endothelial cell, thrombomodulin, primary open-angle glaucoma, exfoliation glaucoma

## Abstract

**Purpose:**

This study aimed to histologically compare the status of Schlemm's canal (SC) and Schlemm's canal endothelial (SCE) cells between trabeculectomy specimens from patients with primary open-angle glaucoma (POAG) and exfoliation glaucoma (EXG).

**Methods:**

A total of 182 eyes from 152 patients with POAG and 138 eyes from 116 patients with EXG underwent immunohistochemical staining for thrombomodulin. Equal numbers of cases were selected from both groups using propensity score matching. The following parameters were evaluated: total SC length, staining positive and negative SC length (PSC and NSC, respectively), opened and closed SC length, staining positive and opened SC length, staining positive and closed SC length, staining negative and opened SC length (NOSC), and staining negative and closed SC length.

**Results:**

After matching for age and gender, 87 cases were selected in each group. The EXG group had significantly higher preoperative IOP and medication scores. PSC was significantly longer in the POAG group, while NSC and NOSC were longer in the EXG group. Multiple regression analysis of these 174 cases revealed that PSC was significantly shorter in the EXG group. After matching for age, gender, preoperative IOP, and medication score, 64 cases were selected in each group, and NOSC was significantly longer in the EXG group.

**Conclusions:**

These findings suggest that in EXG, SCE loss occurs independently of background factors such as aging and medication use. The loss of SCE may have a more critical impact on IOP elevation in EXG compared to POAG.

Glaucoma, with a worldwide estimated prevalence of 3.54% in the population aged 40 to 80 years,^1^ is one of the major causes of severe visual loss and blindness in the world.^2^ The death of retinal ganglion cells (RGCs) and the loss of RGC axons cause glaucomatous optic neuropathy.^3^ Elevated IOP is the primary risk factor for open-angle glaucoma (OAG), including primary open-angle glaucoma (POAG) and glaucoma secondary to pseudoexfoliation syndrome (EXG).^3^ In these OAGs, the increased IOP is explained by reduced aqueous humor outflow from the anterior chamber (AC) into Schlemm's canal (SC), which passes through the trabecular meshwork (TM)/Schlemm's canal endothelium (SCE) complex.^4^

In POAG, the increase of TM resistance is explained by the changes in the amount and quality of the extracellular matrix (ECM) in the TM.^5^ In EXG, an age-related, complex, generalized disorder of the ECM, the progressive accumulation of intraocular abnormal fibrillar materials in the TM is considered the primary cause of chronic IOP elevation.^6^^,^^7^ Although various genetic, internal, and external stress factors, such as immune reactions, inflammation, ischemia, hypoxia, and oxidative stress, have been suggested to be involved,^8^^–^^12^ the precise mechanisms underlying the ECM changes in the TM in OAG are not fully understood.

The size of SC may contribute to increased outflow resistance in SC. In POAG, the cross-sectional area, circumferential length, and inner wall length of SC are significantly smaller than in normal eyes,^13^ and in primary angle closure glaucoma (PACG), when the length of the opened SC is divided into three levels (normal, moderately occluded, and severely occluded), a moderate correlation is observed between SC length and IOP.^14^ Additionally, although not in glaucomatous eyes, it has also been reported that the area of SC on OCT images shows a significant negative correlation with IOP in myopic eyes.^15^ These results suggest that SC morphology correlates with outflow resistance.

Furthermore, even with a normal SC size, the absence of SCEs is associated with SC dysfunction and may contribute to increased IOP.^16^ Aqueous humor was thought to pass through SCEs via micron-sized pores present in the SCEs.^17^^–^^19^ The pore density in SCEs was found to be reduced in glaucomatous eyes compared to normal eyes,^20^^,^^21^ which may lead to increased outflow resistance and elevated IOP.^22^ SCEs are also known to decrease with age.^23^^,^^24^ Among the aged patients with POAG, a remarkably small size in SC was observed in eyes with a family history of glaucoma, while SCE drop-off was significant in eyes without the family history.^25^ These findings suggest that not only SC size but also the number of SCE pores and the presence of SCEs themselves are determinant of IOP.

In previous studies, immunostaining for thrombomodulin (TBM), an inhibitor of platelet aggregation, has been used for histologic examination of SCE.^14^^,^^25^^,^^26^ However, very few studies have conducted a morphologic comparison of SC and SCE between POAG and EXG using anti-TBM antibody staining.

In this study, we focused on SC size and the integrity of SCE. We established a database using trabeculectomy specimens collected in a consecutive series of patients with both POAG and EXG. Background factors were standardized using propensity scores, allowing for a comprehensive comparison of various parameters between the two disease types.

## Patients and Methods

### Patients

This retrospective study adhered to the tenets of the Declaration of Helsinki; the Institutional Review Board (IRB) of Shimane University Hospital reviewed and approved the research conducted at both Shimane University Hospital and Japanese Red Cross Hospital Medical Center (IRB No. 20180703-1; approval date, July 24, 2018). All the patients provided written informed consent prior to the surgery. At the Shimane University Hospital, IRB approval did not require each patient to provide written informed consent for publication; instead, the study protocol was posted at the study institution to opt out the participants from the study. The study included a total of 182 eyes from 152 patients with POAG and 138 eyes from 116 patients with EXG. AC angle tissues were collected during trabeculectomy at the Japanese Red Cross Medical Center between January 1997 and June 2018. All trabeculectomies were performed by one author (TH), and in all patients, angle specimens were obtained from the upper corneoscleral limbus between the 10- and 2-o'clock positions at the time of surgery. For all patients, information of age, sex, maximum preoperative IOP, and preoperative medication score (1 point for each component of topical medication or 1 tablet of oral acetazolamide) was obtained from the sample list.

### Measurement of SC Morphology Parameters

**Figure 1.**
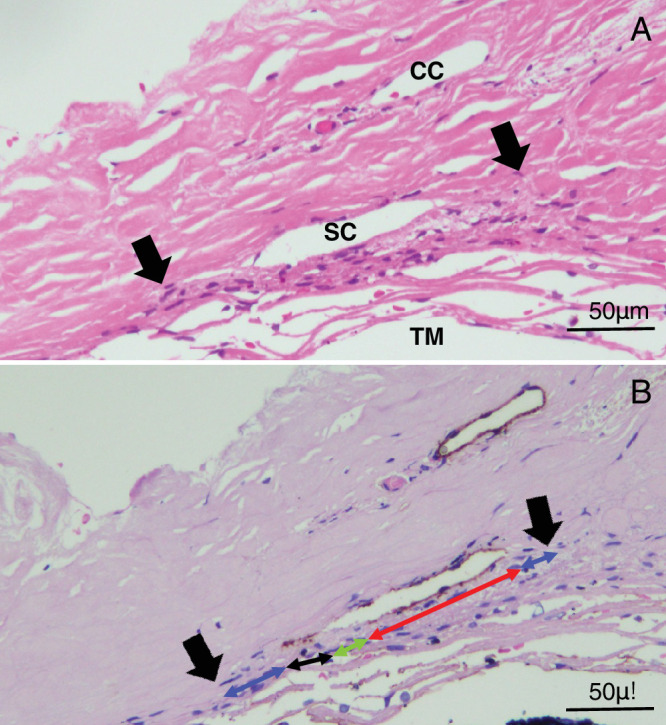
Examples of the SC morphology parameters measurement on specimens stained with hematoxylin and eosin (**A**) and anti-TBM immunohistochemistry (**B**). (**A**) *Black arrows* indicate both ends of the SC. (**B**) *Red double arrow* indicates POSC, *black double arrow* indicates PCSC, *green double arrow* NOSC, and *blue double arrow* indicates NCSC. These specimens are from the POAG eye. CC, collector channel.

**Figure 2.**
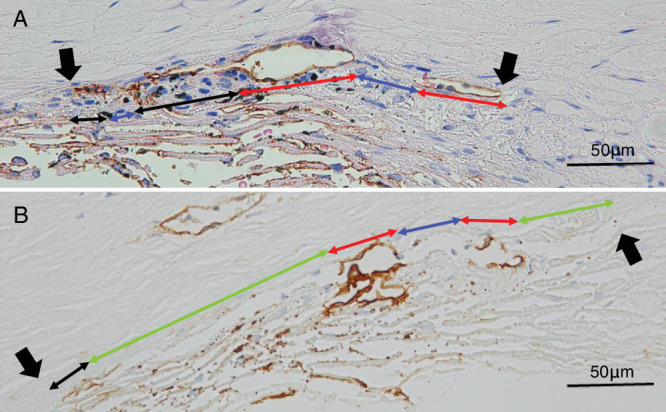
Representative measurement of SC morphology parameters in TBM-stained specimens from patients with POAG (**A**, 79 years old, female) and EXG (**B**, 60 years old, female). (**A**) Collapse of SC (*blue* and *black double arrows*) is dominant in a POAG specimen. (**B**) Drop-off of SC endothelium (*green double arrow*) is dominant in an EXG specimen. *Black arrows* indicate both ends of the SC.

The procedures for trabeculectomy, specimen preparation, and subsequent staining have been described previously.^14^^,^^25^ Briefly, trabeculectomy-excised tissues were fixed overnight in a mixture of 2.5% or 5% formalin and 1% glutaraldehyde, and then they were divided into three to five blocks. All divided specimens were embedded in paraffin and cut into sections with a thickness of 3 µm. These paraffin sections underwent hematoxylin-eosin (HE) staining and immunohistochemical staining with anti-TBM antibodies (clone 1009; Agilent Technologies Japan, Ltd., Tokyo, Japan), an established marker for SCE and collector channel endothelial cells.^14^^,^^25^^,^^27^ The stained sections were then photographed and digitally imaged using an optical microscope system (OLYMPUS BX53, Tokyo, Japan; objective lens ×40, 2448 × 1920 pixels, TIFF format). On the obtained photographs, SC morphology parameters were estimated using ImageJ software (version 1.52a; National Institutes of Health, Bethesda, MD, USA) on a Windows 10 computer. To determine the extent of SC, the anterior and posterior edges were identified in HE-stained images (Fig. 1A, arrows). Four primary parameters were measured on the TBM-stained images depending on the appearance of TBM staining positivity and openings of SC lumen (i.e., presence or absence of SC lumen) (Fig. 1B).^28^ The four primary parameters consisted of the lengths of TBM-positive and opened (POSC, indicated by the red double arrow) or closed (PCSC, the black double arrow) SC, as well as the lengths of TBM-negative and opened (NOSC, the green double arrow) or closed (NCSC, the blue double arow) SC (Figs. 1B, 2A; Table 1). After obtaining these parameters, additional parameters, including the total SC length (TSC, the length between arrows in Figs. 1, 2), TBM positive/negative SC lengths (PSC/NSC), and opened/closed SC lengths (OSC, CSC), were calculated. Definitions of these parameters are listed in Table 1. Owing to the nature of the parameter definitions, alterations in any given parameter are inherently connected to changes in the corresponding other parameters. All the images were evaluated independently by two examiners (i.e., AT and TH), and the four primary parameters were determined by consensus between the examiners. Out of the sections analyzed, 26 eyes in the POAG group and 5 eyes in the EXG group were excluded from the study due to tissue destruction during excision of the TM.

**Table 1.** Definition of Parameters

Abbreviation	Parameter Name	Definition
TSC	Total SC length	POSC + PCSC + NOSC + NCSC
PSC	TBM positive SC length	POSC + PCSC
NSC	TBM negative SC length	NOSC + NCSC
OSC	Opened SC length	POSC + NOSC
CSC	Closed SC length	PCSC + NCSC
POSC	TBM positive and opened SC length	Length of TBM positive and opened SC on TBM staining
PCSC	TBM positive and closed SC length	Length of TBM positive and closed SC on TBM staining
NOSC	TBM negative and opened SC length	Length of TBM negative and opened SC on TBM staining
NCSC	TBM negative and closed SC length	Length of TBM negative and closed SC on TBM staining

### Statistical Analysis

To compare the measured parameters between the POAG and EXG groups, an equal number of eyes were selected from each group using propensity score matching with the nearest available matching method (random number seed value = 6,680,040, caliper coefficient = 0.20). Propensity score matching was conducted based on two covariates (i.e., age and gender; *n* = 87 in each group) or on four covariates (i.e., age, gender, maximum preoperative IOP, and medication score; *n* = 64 in each group). After selecting the cases, the measurement parameters were compared between the POAG and EXG groups by unpaired *t*-test for continuous variables and by Fisher's exact probability test for categorical variables. The possible association between the measured parameters and background parameters was assessed by multiple regression analyses. A *P* value less than 0.05 was considered statistically significant. All statistical analyses were performed using the JMP Pro statistical software version 16.00 (SAS Institute, Inc., Cary, NC, USA).

## Results

When matched for age and gender, 87 cases each were selected in each group. Patient background factors are shown in Table 2. In these cases, compared with POAG, the maximum preoperative IOP (*P* < 0.0001) and medication score (*P* = 0.0027) were significantly higher in the EXG group (Table 2). Table 3 shows the comparison of SC morphology parameters between the two disease groups. The TSC was similar between the POAG (287.1 µm) and EXG (300.5 µm) groups (*P* = 0.16), while the PSC (*P* = 0.025) was significantly shorter and, vice versa, NSC (*P* = 0.0010) and NOSC (*P* < 0.0001) were significantly longer in the EXG group than the POAG group (Table 3). This suggests a reduced presence of TBM-positive SCE in the EXG, despite the maintenance of the SC lumen.

**Table 2.** Demographic Data of Age- and Gender-Matched POAG and EXG Groups

Characteristic	POAG (*n* = 87)	EXG (*n* = 87)	*P* Value
Age, y			
Mean ± SD	71.0 ± 6.7	71.2 ± 6.8	0.87
Range	54 to 85	54 to 85	
95% CI	69.6 to 72.5	69.8 to 72.7	
Gender, *n* (%)			
Male	48 (55.2)	42 (48.3)	0.45
Female	39 (44.8)	45 (51.7)	
Preoperative IOP, mm Hg			
Mean ± SD	23.7 ± 6.7	31.1 ± 9.3	<0.0001^*^
Range	8 to 53	17 to 54	
95% CI	22.3 to 25.1	29.0 to 33.2	
Preoperative medications			
Mean ± SD	3.7 ± 1.2	4.3 ± 1.5	0.0027^*^
Range	1 to 6	1 to 9	
95% CI	3.4 to 3.9	4.0 to 4.7	
Eye, *n* (%)			
Right	48 (55.8)	38 (44.2)	0.17
Left	38 (44.2)	48 (55.8)	

Continuous variables are compared by *t*-test, and categorical variables are compared by Fisher's exact probability test.

* Indicates a significance level of 1% (*P* < 0.01).

**Table 3.** Comparisons of Measured Parameters Between Age- and Gender-Matched POAG and EXG Groups

Characteristic	POAG (*n* = 87)	EXG (*n* = 87)	*P* Value
TSC (µm)			
Mean ± SD	287.1 ± 48.0	300.5 ± 74.4	0.16
Range	160.0 to 401.2	182.2 to 602.2	
95% CI	276.9 to 297.3	284.6 to 316.4	
PSC (µm)			
Mean ± SD	164.0 ± 71.3	136.9 ± 86.2	0.025^*^
Range	0 to 332.0	0 to 494.4	
95% CI	148.8 to 179.2	118.5 to 155.3	
NSC (µm)			
Mean ± SD	123.0 ± 75.0	163.6 ± 84.7	0.0010^**^
Range	0 to 365.2	0 to 435.4	
95% CI	107.1 to 139.0	145.6 to 181.7	
OSC (µm)			
Mean ± SD	191.7 ± 84.0	220.9 ± 125.9	0.073
Range	29.4 to 374.5	0 to 602.2	
95% CI	173.8 to 209.6	194.1 to 247.8	
CSC (µm)			
Mean ± SD	95.4 ± 79.0	79.6 ± 87.6	0.21
Range	0 to 270.2	0 to 299.7	
95% CI	79.0 to 111.8	60.9 to 98.2	
POSC (µm)			
Mean ± SD	140.1 ± 70.1	122.1 ± 88.4	0.14
Range	0 to 293.7	0 to 494.4	
95% CI	125.1 to 155.0	103.3 to 141.0	
PCSC (µm)			
Mean ± SD	24.0 ± 34.1	14.8 ± 28.2	0.053
Range	0 to 202.0	0 to 142.3	
95% CI	16.8 to 31.3	8.8 to 20.8	
NOSC (µm)			
Mean ± SD	51.7 ± 58.8	98.8 ± 92.4	<0.0001^**^
Range	0 to 337.4	0 to 435.4	
95% CI	39.1 to 64.2	79.1 to 118.5	
NCSC (µm)			
Mean ± SD	71.4 ± 70.2	64.8 ± 80.4	0.57
Range	0 to 265.4	0 to 299.7	
95% CI	56.4 to 86.3	47.7 to 81.9	

*P* values are calculated by t-test.

* Significance levels of 5% (*P* < 0.05).

** Significance levels of 1% (*P* < 0.01).

To assess the potential effects of background factors on the morphology parameters, multiple regression analyses were performed, including age, gender, maximum preoperative IOP, medication score, and disease type as dependent variables in these 174 cases (Table 4). The results showed that older age was associated with longer NSC (*P* = 0.0053) and NCSC (*P* = 0.033), while it was associated with shorter PSC (*P* < 0.0001), OSC (*P* = 0.031), and POSC (*P* = 0.0001). Female gender was linked to shorter POSC (*P* = 0.030), and a higher medication score was associated with longer CSC (*P* = 0.011) and NCSC (*P* = 0.011). Even after adjusting for differences in background factors by this multivariate analysis, once again, EXG rather than POAG was associated with longer NOSC (*P* = 0.0012).

**Table 4.** Multivariate Analysis in Age- and Gender-Matched POAG and EXG Groups

Characteristic	Age, y	Gender, Female/Male	Preoperative IOP, mm Hg	Preoperative Medications, Component	Disease, EXG/POAG
TSC					
*r*	−0.81	−4.91	1.01	1.54	2.91
95% CI	−2.1 to 0.5	−13.8 to 4.0	−0.1 to 2.1	−5.0 to 8.1	−6.9 to 12.7
*P* value	0.22	0.28	0.07	0.64	0.56
PSC					
*r*	−3.4	−7.29	−0.38	−4.47	−9.7
95% CI	−5.0 to −1.8	−18.4 to 3.8	−1.7 to 1.0	−12.7 to 3.7	−21.9 to 2.5
*P* value	<0.0001^**^	0.19	0.59	0.28	0.12
NSC					
*r*	2.58	2.39	1.39	6.01	12.61
95% CI	0.8 to 4.4	−9.8 to 14.5	−0.1 to 2.9	−3.0 to 15.0	−0.8 to 26.0
*P* value	0.0053^**^	0.70	0.07	0.19	0.06
OSC					
*r*	−2.56	−11.4	0.88	−10.71	16.79
95% CI	−4.9 to −0.2	−27.1 to 4.3	−1.1 to 2.8	−22.3 to 0.9	−0.5 to 34.1
*P* value	0.031^*^	0.15	0.37	0.07	0.06
CSC					
*r*	1.75	6.49	0.13	12.25	−13.88
95% CI	−0.1 to 3.6	−6.2 to 19.2	−1.4 to 1.7	2.9 to 21.6	−27.9 to 0.1
*P* value	0.07	0.31	0.87	0.011*	0.052
POSC					
*r*	−3.28	−12.13	0.05	−5.65	−5.55
95% CI	−4.9 to −1.7	−23.1 to −1.2	−1.3 to 1.4	−13.7 to 2.4	−17.6 to 6.5
*P* value	0.0001^**^	0.030^*^	0.95	0.17	0.36
PCSC					
*r*	−0.12	4.83	−0.42	1.18	−4.16
95% CI	−0.8 to 0.6	−0.0 to 9.7	−1.0 to 0.2	−2.4 to 4.8	−9.5 to 1.2
*P* value	0.74	0.05	0.16	0.52	0.13
NOSC					
*r*	0.72	0.73	0.83	−5.06	22.34
95% CI	−1.1 to 2.5	−11.4 to 12.8	−0.7 to 2.3	−14.0 to 3.9	9.0 to 35.7
*P* value	0.43	0.91	0.27	0.27	0.0012^**^
NCSC					
*r*	1.87	1.66	0.56	11.07	−9.73
95% CI	0.2 to 3.6	−9.9 to 13.2	−0.9 to 2.0	2.5 to 19.6	−22.4 to 3.0
*P* value	0.033^*^	0.78	0.44	0.011^*^	0.13

*P* and *r* values are calculated by multiple regression analysis.

* Significance levels of 5% (*P* < 0.05).

** Significance levels of 1% (*P* < 0.01).

To further adjust for background factors, the cases were matched by four covariates (Table 5) and compared the SC morphology parameters between disease group (Table 6). The results indicated that NOSC (*P* = 0.0049) was significantly longer in EXG than POAG (Table 6). Figure 2 shows the representative TBM immunostaining from both disease groups. In the POAG specimen, collapse of SC (indicated by blue and black double arrows) was dominant (Fig. 2A), while drop-off of SCE, despite the presence of SC lumen (indicated by green double arrow), was dominant in the EXG specimen (Fig. 2B).

**Table 5.** Demographic Data of Age-, Gender-, Preoperative IOP-, and Medications-Matched POAG and EXG Groups

Characteristic	POAG (*n* = 64)	EXG (*n* = 64)	*P* Value
Age, y			
Mean ± SD	72.7 ± 6.7	71.3 ± 7.1	0.26
Range	44 to 85	54 to 93	
95% CI	71.0 to 74.3	69.5 to 73.0	
Gender, *n* (%)			0.72
Male	39 (60.9)	36 (56.3)	
Female	25 (39.1)	28 (43.8)	
Preoperative IOP, mm Hg			
Mean ± SD	25.9 ± 6.8	27.7 ± 7.8	0.15
Range	12 to 53	17 to 54	
95% CI	24.2 to 27.6	25.8 to 29.7	
Preoperative medications			
Mean ± SD	3.9 ± 1.2	3.9 ± 1.5	0.90
Range	1 to 6	1 to 7	
95% CI	3.6 to 4.2	3.5 to 4.3	
Eye, *n* (%)			
Right	36 (56.3)	28 (44.4)	0.22
Left	28 (43.8)	35 (55.6)	

Continuous variables are compared by *t*-test, and categorical variable are compared by Fisher's exact probability test.

**Table 6.** Comparisons of Measured Parameters Between Age-, Gender-, Preoperative IOP-, and Medications-Matched POAG and EXG Groups

Characteristic	POAG (*n* = 64)	EXG (*n* = 64)	*P* Value
TSC (µm)			
Mean ± SD	288.9 ± 51.0	289.3 ± 54.8	0.96
Range	160.0 to 401.2	183.1 to 448.8	
95% CI	276.1 to 301.6	275.7 to 303.0	
PSC (µm)			
Mean ± SD	160.1 ± 71.3	135.8 ± 68.0	0.05
Range	0 to 293.7	0 to 264.4	
95% CI	142.3 to 177.9	118.8 to 152.8	
NSC (µm)			
Mean ± SD	128.8 ± 79.4	153.5 ± 79.9	0.08
Range	0 to 365.2	0 to 371.3	
95% CI	108.9 to 148.6	133.6 to 173.5	
OSC (µm)			
Mean ± SD	188.5 ± 90.9	207.7 ± 105.9	0.27
Range	29.4 to 374.5	0 to 448.8	
95% CI	165.8 to 211.2	181.3 to 234.2	
CSC (µm)			
Mean ± SD	100.4 ± 77.1	81.6 ± 87.4	0.20
Range	0 to 270.2	0 to 299.7	
95% CI	81.1 to 119.6	59.8 to 103.4	
POSC (µm)			
Mean ± SD	139.1 ± 73.7	120.2 ± 71.2	0.14
Range	0 to 293.7	0 to 264.4	
95% CI	120.7 to 157.5	102.4 to 138.0	
PCSC (µm)			
Mean ± SD	21.0 ± 23.5	15.6 ± 28.9	0.24
Range	0 to 103.8	0 to 142.3	
95% CI	15.2 to 26.9	8.4 to 22.8	
NOSC (µm)			
Mean ± SD	49.4 ± 65.8	87.5 ± 83.7	0.0049^**^
Range	0 to 337.4	0 to 310.9	
95% CI	33.0 to 65.9	66.6 to 108.4	
NCSC (µm)			
Mean ± SD	79.3 ± 69.7	66.0 ± 79.0	0.31
Range	0 to 265.4	0 to 299.7	
95% CI	61.9 to 96.8	46.3 to 85.8	

*P* values are calculated by *t*-test.

** Indicates significance levels of 1% (*P* < 0.01).

Previously, Hamanaka et al.^25^ proposed the “percentage of the TBM-negative area of SC (PTNA)” as a parameter for assessing SCE drop-off. To compare these previous results, additional parameters, including PTNA, were further defined (Supplementary Table S1)^28^ and compared between the two-covariate (Supplementary Table S2) and four-covariate (Supplementary Table S3) matched disease groups. The results indicated that %Nin OSC, which is equivalent to Hamanaka's PTNA, was significantly larger in EXG than POAG in both match data sets.

## Discussion

In this study, we examined the morphology of SC in trabeculectomy specimens from patients with POAG and EXG. We conducted a comparative analysis between background-matched disease groups, setting a unique and thorough approach by using propensity score matching on a large cohort of patients with OAG. Although POAG and EXG are representative forms of OAG, our study unveiled differences in SC morphology parameters between these two disease types.

When matched for age and gender, the EXG group exhibited significantly higher maximum preoperative IOP and a greater medication score as background factors. Regarding SC morphologic parameters, the TSC showed no significant differences between the two groups. However, the PSC, representing segments positive for TBM, was significantly longer in POAG, while the length of TBM-negative and opened SC (NOSC) was notably longer in EXG. In our previous study, we demonstrated that the staining results with various SCE markers (such as thrombomodulin, CD31, and CD34) closely matched those in serial sections.^28^ Furthermore, we have previously confirmed the disappearance of SCE in areas of negative staining using transmission electron microscopy.^25^ Therefore, we assert that a negative stain with these SCE markers is indicative of the loss of SCE, rather than merely reflecting a reduced expression of these markers. These findings led us to hypothesize that the loss of SCE might be more prominent in EXG compared to POAG. Multivariate analysis confirmed that maximal preoperative IOP and medication score were also influential factors contributing to the drop-off of SCE and narrowing of SC. Therefore, we performed propensity score matching again, now including maximum preoperative IOP and medication score, in addition to age and gender. This time, the TSC measurements were 288.9 µm for POAG and 289.3 µm for EXG, with no statistically significant difference. In a previous study, Allingham et al.^13^ reported an average TSC in normal eyes to be 264 ± 55 µm, in a group with a mean age of 73.2 years, and the TSCs in both the POAG and EXG groups in our study appeared to be within the normal range. Furthermore, when matched for age, gender, maximum preoperative IOP, and medication score, NOSC was significantly longer in the EXG group, indicating a greater drop-off in SCE and more open SC lumens in EXG compared to POAG, independent of background factors. These findings lend support to the hypothesis that EXG is associated with a more pronounced loss of SCE.

In the multivariate analysis, we observed a decrease in PSC, OSC, and POSC, as well as an increase in NSC and NCSC with aging. These results suggest that aging is associated with both SCE drop-off and a reduction in SC size. The drop-off of SCE with aging has previously been reported in normal eyes and in POAG, especially in cases with a family history of the condition.^23^^,^^25^ Interestingly, we also found a correlation between female gender and the shortening of POSC, although the reasons for this relationship remain unclear and warrant further research. A higher medication score was found to be associated with longer CSC and NCSC. Since a high medication score typically indicates high IOP, this correlation likely reflects the influence of preoperative IOP. However, the direct impact of the eye drops themselves cannot be ruled out. Johnson and Matsumoto^29^ reported that SC tended to narrow after filtration surgery in POAG eyes, likely due to aqueous humor bypassing the TM and SC, draining into the bleb, thus reducing outflow into the main pathway. We have previously reported cases of choroidal detachment following microhook trabeculotomy, in which IOP increased after the resolution of choroidal detachment probably due to the reduced main pathway function during the hypotony.^30^ Furthermore, Okuda et al.^31^ recently documented that preoperative use of lipasudil, known to promote main outflow pathway, improved the surgical outcomes of microhook trabeculotomy. These observations suggest that diminished SC function may result from inadequate perfusion of the main outflow pathway. Gabelt and Kaufman^32^ reported a reduction of aqueous humor outflow through the main pathway by one-third in a group using prostaglandin eye drops compared to a control group in studies involving monkeys. Prostaglandin eye drops, known to promote uveoscleral outflow, could potentially affect the main outflow pathway through a similar mechanism. Given the likelihood that many of the patients in our study were using prostaglandin eye drops, it is essential to acknowledge that the specific types of eye drops used were not documented in this study. Future research should consider including information about the specific eye drop formulations to gain a more comprehensive understanding of their potential effects on SC and IOP regulation.

In EXG, IOP increase is attributed to the accumulation of abnormal fibrillar materials within the aqueous humor outflow tract, particularly in the subendothelial region of SC.^33^^,^^34^ The synthesis of abnormal fibrillar materials in EXG may be driven by fibrogenic growth factors (e.g., TGF-β), cytokines (e.g., IL-6), amino acids (e.g., homocysteine), and various stress conditions such as oxidative stress, ultraviolet radiation, and hypoxia.^35^^,^^36^ SCE drop-off itself is not unique to EXG and can also be observed in inflammatory diseases like atopic dermatitis and glaucoma secondary to uveitis.^37^^,^^38^ In addition, patients with EXG show an increase in advanced glycation end products (AGEs), associated with heightened oxidative stress,^39^^,^^40^ further indicating the potential role of inflammation and increased oxidative stress in SCE loss in EXG. Aging was found to result in both SCE drop-off and a shortening of OSC, but in EXG, only SCE drop-off was observed, suggesting that EXG may not solely be an age-related alteration but may instead indicate some form of damage or impairment specifically affecting the SCE. The inner wall of SCE is considered to comprise the blood–aqueous barrier (BAB) within the eye, along with the nonpigmented epithelium of the ciliary process and the endothelial cells in the iris vasculature. Therefore, the decrease in SCE in EXG may explain BAB impairment in EXG,^41^ a topic worth further investigation in the future.

In contrast, POAG is a type of glaucoma in which SCE typically does not drop off but SC tends to become smaller. Abnormal SC development has been reported in primary congenital glaucoma (PCG)^42^ and juvenile open-angle glaucoma (JOAG).^43^ With regard to SC size, it has been reported that PCG often shows smaller SC or even the absence of SC,^44^ POAG with family history of POAG has smaller SC,^25^ and JOAG has smaller SC than older-onset POAG without family history.^28^ Although the clinical boundaries between PCG, JOAG, and POAG may sometimes be ambiguous, our present study found no correlation between age and TSC, suggesting that some cases of POAG may involve congenital SC developmental abnormalities.

This study stands out for its unique approach, analyzing a large number of consecutive cases of POAG and EXG using propensity score matching. Notably, the analysis was carried out on living human eye tissues rather than relying on cadaveric eyes. However, certain limitations should be acknowledged. First, this study primarily included cases where medications and other conventional treatments had proven ineffective, leading to a predominance of severe cases (typically, stage IV or higher according to the Aulhorn–Greve classification in both groups) that were suitable for trabeculectomy. Regarding the dates of surgery, the POAG group's samples were collected from 2014 to 2019, while those from the EXG group spanned from 1997 to 2018. This discrepancy arose from the different numbers of prematching samples available for each group. Nevertheless, we believe that this age difference does not affect the results, as the methods for tissue collection and processing have been consistent since 1997. Additionally, only a small segment of SC tissue was analyzed, which might not capture the full extent of morphologic changes in the canal. Another limitation is the absence of data regarding visual fields, fundus changes, or preoperative AC angle findings. Furthermore, the possibility that the type of eye drops used may affect SC and SCE morphology cannot be ruled out. The absence of a comparison with normal eyes is also a limitation, but obtaining tissue from normal eyes is a challenging issue.

In conclusion, our study revealed that SCE drop-off occurred independent of background factors such as aging and medication use in EXG. This suggests that SCE loss may contribute more critically to IOP elevation in EXG than in POAG.

## Supplementary Material

Supplement 1
