# Supplementary material for: Comparison of Schlemm's Canal Morphology Parameters Between Propensity Score–Matched Primary Open-Angle Glaucoma and Exfoliation Glaucoma
Source: Invest Ophthalmol Vis Sci. 2024 Feb 7;65(2):15. doi: 10.1167/iovs.65.2.15 (PMC10854412; doi:10.1167/iovs.65.2.15)
Supplement: Supplement 1 [file iovs-65-2-15_s001.pdf]

Table S1. Aditional definitions of parameters

Parameters	Definition
%PSC	TBM positive Schlemm's canal length / Total Schlemm's canal length × 100
%NSC	TBM negative Schlemm's canal length / Total Schlemm's canal length × 100
%OSC	Opened Schlemm's canal length / Total Schlemm's canal length × 100
%CSC	Closed Schlemm's canal length / Total Schlemm's canal length × 100
%POSC	TBM positive and opened Schlemm's canal length / Total Schlemm's canal length × 100
%PCSC	TBM positive and closed Schlemm's canal length / Total Schlemm's canal length × 100
%NOSC	TBM negative and opened Schlemm's canal length / Total Schlemm's canal length × 100
%NCSC	TBM negative and closed Schlemm's canal length / Total Schlemm's canal length × 100
%OinPSC	TBM positive and opened Schlemm's canal length / TBM positive Schlemm's canal length × 100
%CinPSC	TBM positive and closed Schlemm's canal length / TBM positive Schlemm's canal length × 100
%OinNSC	TBM negative and opened Schlemm's canal length / TBM negative Schlemm's canal length × 100
%CinNSC	TBM negative and closed Schlemm's canal length / TBM negative Schlemm's canal length × 100
%PinOSC	TBM positive and opened Schlemm's canal length / Opened Schlemm's canal length × 100
%NinOSC	TBM negative and opened Schlemm's canal length / Opened Schlemm's canal length × 100
%PinCSC	TBM positive and closed Schlemm's canal length / Closed Schlemm's canal length × 100
%NinCSC	TBM negative and closed Schlemm's canal length / Closed Schlemm's canal length × 100

TBM, thrombomudulin immuno-staining

Table S2. Comparisons of measured parameters between age- and gender-matched POAG and EXG groups

		POAG (n=87)	EXG (n=87)	p-value
%PSC (%)				
	Mean ± SD	57.6 ± 23.1	46.0 ± 24.7	0.0017**
	Range	0, 100	0, 100	
	95% CI	52.7, 62.5	40.8, 51.3	
%NSC (%)				
	Mean ± SD	42.4 ± 23.1	54.0 ± 24.7	0.0017**
	Range	0, 100	0, 100	
	95% CI	37.5, 47.3	48.7, 59.2	
%OSC (%)				
	Mean ± SD	66.3 ± 26.0	71.0 ± 31.4	0.28
	Range	9.5, 100	0, 100	
	95% CI	60.8, 71.8	64.3, 77.7	
%CSC (%)				
	Mean ± SD	33.7 ± 26.0	29.0 ± 31.4	0.28
	Range	0, 90.5	0, 100	
	95% CI	28.2, 39.2	22.3, 35.7	
%POSC (%)				
	Mean ± SD	48.9 ± 23.3	40.6 ± 24.8	0.023*
	Range	0, 100	0, 91.2	
	95% CI	44.0, 53.9	35.3, 45.9	
%PCSC (%)				
	Mean ± SD	8.7 ± 11.7	5.4 ± 10.5	0.057
	Range	0, 57.3	0, 47.9	
	95% CI	6.2, 11.2	3.2, 7.7	
%NOSC (%)				
	Mean ± SD	17.4 ± 18.9	30.3 ± 26.0	0.0002**
	Range	0, 92.7	0, 100	
	95% CI	13.3, 21.4	24.8, 35.9	
%NCSC (%)				
	Mean ± SD	25.0 ± 23.4	23.6 ± 28.6	0.73
	Range	0, 85.4	0, 100	
	95% CI	20.0, 30.0	17.5, 29.7	
%OinPSC (%)				
	Mean ± SD	81.6 ± 23.1	80.9 ± 31.1	0.87
	Range	0, 100	0, 100	
	95% CI	76.6, 86.5	74.2, 87.5	
%CinPSC (%)				
	Mean ± SD	16.0 ± 19.8	11.1 ± 20.5	0.11
	Range	0, 72.2	0, 100	
	95% CI	11.7, 20.2	6.7, 15.5	
%OinNSC (%)				

	Mean ± SD	39.9 ± 35.4	56.5 ± 40.9	0.0048**
	Range	0, 100	0, 100	
	95% CI	32.3, 47.4	47.8, 65.2	
%CinNSC (%)				
	Mean ± SD	52.8 ± 35.8	40.4 ± 40.2	0.033*
	Range	0, 100	0, 100	
	95% CI	45.2, 60.5	31.9, 49.0	
%PinOSC (%)				
	Mean ± SD	77.2 ± 23.5	59.2 ± 28.5	<0.0001**
	Range	0, 100	0, 100	
	95% CI	72.2, 82.2	53.2, 65.3	
%NinOSC (%)				
	Mean ± SD	19.7 ± 20.7	34.4 ± 27.2	<0.0001**
	Range	0, 100	0, 100	
	95% CI	15.3, 24.1	28.7, 40.2	
%PinCSC (%)				
	Mean ± SD	20.8 ± 23.0	12.3 ± 23.6	0.017*
	Range	0, 100	0, 100	
	95% CI	15.9, 25.8	7.3, 17.3	
%NinCSC (%)				
	Mean ± SD	50.8 ± 32.3	45.0 ± 40.3	0.29
	Range	0, 100	0, 100	
	95% CI	43.9, 57.7	36.4, 53.5	

P-values are calculated by t-test. * and ** indicate significance levels of 5% ($p<0.05$) and 1% ($p<0.01$), respectively.

%PSC, TBM positive Schlemm's canal length / Total Schlemm's canal length × 100; %NSC, TBM negative Schlemm's canal length / Total Schlemm's canal length × 100; %OSC, Opened Schlemm's canal length / Total Schlemm's canal length × 100; %CSC, Closed Schlemm's canal length / Total Schlemm's canal length × 100; %POSC, TBM positive and opened Schlemm's canal length / Total Schlemm's canal length × 100; %PCSC, TBM positive and closed Schlemm's canal length / Total Schlemm's canal length × 100; %NOSC, TBM negative and opened Schlemm's canal length / Total Schlemm's canal length × 100; %NCSC, TBM negative and closed Schlemm's canal length / Total Schlemm's canal length × 100; %OinPSC, TBM positive and opened Schlemm's canal length / TBM positive Schlemm's canal length × 100; %CinPSC, TBM positive and closed Schlemm's canal length / TBM positive Schlemm's canal length × 100; %OinNSC, TBM negative and opened Schlemm's canal length / TBM negative Schlemm's canal length × 100; %CinNSC, TBM negative and closed Schlemm's canal length / TBM negative Schlemm's canal length × 100; %PinOSC, TBM positive and opened Schlemm's canal length / Opened Schlemm's canal length × 100; %NinOSC, TBM negative and opened Schlemm's canal length / Opened Schlemm's canal length × 100; %PinCSC, TBM positive and closed Schlemm's canal length / Closed Schlemm's canal length × 100; %NinCSC, TBM negative and closed Schlemm's canal length / Closed Schlemm's canal length × 100; POAG, primary open angle glaucoma; EXG, exfoliation glaucoma; SD, standard deviation.

Table S3. Comparisons of measured parameters between age-, gender-, preoperative IOP-, and medications-matched POAG and EXG groups

		POAG (n=64)	EXG (n=64)	p-value
%PSC (%)				
	Mean ± SD	56.6 ± 23.8	48.3 ± 24.2	0.051
	Range	0, 100	0, 100	
	95% CI	50.7, 62.6	42.2, 54.3	
%NSC (%)				
	Mean ± SD	43.4 ± 23.8	51.7 ± 24.2	0.051
	Range	0, 100	0, 100	
	95% CI	37.4, 49.3	45.7, 57.8	
%OSC (%)				
	Mean ± SD	64.1 ± 26.3	71.0 ± 30.5	0.17
	Range	9.5, 100	0, 100	
	95% CI	57.5, 70.6	63.3, 78.6	
%CSC (%)				
	Mean ± SD	35.9 ± 26.3	29.0 ± 30.5	0.17
	Range	0, 90.5	0, 100	
	95% CI	29.4, 42.5	21.4, 36.7	
%POSC (%)				
	Mean ± SD	48.8 ± 24.4	42.8 ± 25.0	0.17
	Range	0, 100	0, 91.2	
	95% CI	42.7, 54.9	36.5, 49.0	
%PCSC (%)				
	Mean ± SD	7.8 ± 9.1	5.5 ± 10.0	0.16
	Range	0, 39.4	0, 47.9	
	95% CI	5.6, 10.1	3.0, 8.0	
%NOSC (%)				
	Mean ± SD	15.3 ± 19.1	28.3 ± 25.3	0.0014**
	Range	0, 92.7	0, 87.0	
	95% CI	10.5. 20.0	21.9, 34.6	
%NCSC (%)				
	Mean ± SD	28.1 ± 23.4	23.5 ± 27.5	0.31
	Range	0, 85.4	0, 100	
	95% CI	22.3, 34.0	16.6, 30.4	
%OinPSC (%)				
	Mean ± SD	80.9 ± 22.3	83.8 ± 28.0	0.52
	Range	0, 100	0, 100	
	95% CI	75.3, 86.4	76.8, 90.8	
%CinPSC (%)				
	Mean ± SD	16.3 ± 19.3	11.6 ± 21.0	0.19
	Range	0, 72.2	0, 100	
	95% CI	11.4, 21.1	6.3, 16.8	

%OinNSC (%)				
	Mean ± SD	34.6 ± 33.5	53.3 ± 41.0	0.0054**
	Range	0, 100	0, 100	
	95% CI	26.2, 43.0	43.1, 63.6	
%CinNSC (%)				
	Mean ± SD	59.4 ± 34.0	42.5 ± 40.1	0.011*
	Range	0, 100	0, 100	
	95% CI	50.9, 67.9	32.5, 52.5	
%PinOSC (%)				
	Mean ± SD	78.7 ± 23.5	64.1 ± 30.0	0.0026**
	Range	0, 100	0, 100	
	95% CI	72.9, 84.6	56.6, 71.6	
%NinOSC (%)				
	Mean ± SD	17.6 ± 20.8	32.8 ± 28.3	0.0008**
	Range	0, 100	0, 100	
	95% CI	12.4, 22.8	25.7, 39.8	
%PinCSC (%)				
	Mean ± SD	17.5 ± 18.5	13.5 ± 24.5	0.31
	Range	0, 73.6	0, 100	
	95% CI	12.8, 22.1	7.4, 19.7	
%NinCSC (%)				
	Mean ± SD	56.5 ± 32.0	47.4 ± 39.8	0.16
	Range	0, 100	0, 100	
	95% CI	48.5, 64.5	37.5, 57.3	

P-values are calculated by t-test. * and ** indicate significance levels of 5% ($p<0.05$) and 1% ($p<0.01$), respectively.

%PSC, TBM positive Schlemm's canal length / Total Schlemm's canal length × 100; %NSC, TBM negative Schlemm's canal length / Total Schlemm's canal length × 100; %OSC, Opened Schlemm's canal length / Total Schlemm's canal length × 100; %CSC, Closed Schlemm's canal length / Total Schlemm's canal length × 100; %POSC, TBM positive and opened Schlemm's canal length / Total Schlemm's canal length × 100; %PCSC, TBM positive and closed Schlemm's canal length / Total Schlemm's canal length × 100; %NOSC, TBM negative and opened Schlemm's canal length / Total Schlemm's canal length × 100; %NCSC, TBM negative and closed Schlemm's canal length / Total Schlemm's canal length × 100; %OinPSC, TBM positive and opened Schlemm's canal length / TBM positive Schlemm's canal length × 100; %CinPSC, TBM positive and closed Schlemm's canal length / TBM positive Schlemm's canal length × 100; %OinNSC, TBM negative and opened Schlemm's canal length / TBM negative Schlemm's canal length × 100; %CinNSC, TBM negative and closed Schlemm's canal length / TBM negative Schlemm's canal length × 100; %PinOSC, TBM positive and opened Schlemm's canal length / Opened Schlemm's canal length × 100; %NinOSC, TBM negative and opened Schlemm's canal length / Opened Schlemm's canal length × 100; %PinCSC, TBM positive and closed Schlemm's canal length / Closed Schlemm's canal length × 100; %NinCSC, TBM negative and closed Schlemm's canal length / Closed Schlemm's canal length × 100; POAG, primary open angle glaucoma; EXG, exfoliation glaucoma; SD, standard deviation.
